# Challenges for gene therapy in the financial sustainability of health systems: a scoping review

**DOI:** 10.1186/s13023-024-03249-z

**Published:** 2024-06-24

**Authors:** Hugo Ossandon, Nicolás Armijo, Constanza Vargas, Gabriela M. Repetto, Manuel Antonio Espinoza

**Affiliations:** 1 Departamento de Coordinación de Garantías y Prestaciones de Salud, División de Planificación Sanitaria, Ministerio de Salud de Chile, Enrique McIver 421, Santiago, Chile; 2 Centro de Investigación Clínica, Unidad de Evaluación de Tecnologías en Salud (ETESA UC), Pontificia Universidad Católica de Chile, Santiago, Chile; 3 Centre for Health Economics Research and Evaluation (CHERE), University of Technology Sydney, Sydney, Australia; 4 Rare Diseases Program, Institute of Science and Innovation in Medicine, Facultad de Medicina, Clinica Alemana Universidad del Desarrollo, Santiago, Chile; 5 Centro Para la Prevención y Control del cáncer, Santiago, Chile; 6 Departamento de Salud Pública, Pontificia Universidad Católica de Chile, Santiago, Chile

## Abstract

**Aim:**

To review the available evidence about the strategies implemented or proposed for coverage or reimbursement for currently approved gene therapies.

**Methods:**

A scoping review was conducted to analyze the evidence published during the years 2016 to 2023. The main search criteria were coverage or reimbursement of gene therapy by healthcare systems. The eligible articles were those that described or proposed a financing model used to provide coverage in the various systems around the world.

**Results:**

The study identified 279 publications, and after removing duplicates and screening for eligibility, 10 were included in the study. The results show that various financing models have been proposed, including subscription-based payment models, outcome-based payment models, and amortization strategies. However, several barriers to implementing these models were identified, such as deficiencies in informatics systems for data collection, changes in laws or regulations, the lack of accessible clinical endpoints and administrative costs.

**Conclusion:**

This scoping review provides an overview of financing strategies for gene therapies. Gene therapies can cure rare or previously intractable diseases, but their high cost can make access difficult. Publishing experiences with these models can help evaluate their use and gather more evidence for their effectiveness.

**Supplementary Information:**

The online version contains supplementary material available at 10.1186/s13023-024-03249-z.

## Background

Gene therapy is a technique to treat and prevent diseases by adding a new gene or replacing or repairing an altered gene [1, 2]. These advances hold great hope for treating some devastating rare and inherited conditions and incurable diseases [3, 4]. Understanding the precise pathogenic mechanisms of diseases which can then lead to the development of specific and efficient gene selection and delivery tools, is expected to revolutionize disease treatment and the pharmaceuticals market [3, 4].

One challenge associated with gene therapy is the limited accessibility for patients. This issue primarily arises from the substantial research and development costs borne by academia, pharmaceutical companies and others in the creation of these medical technologies, as well as the anticipation of significant returns of investment upon commercialization [5]. Furthermore, these therapies target a small population of individuals afflicted by rare or ultra-rare diseases [6]. Consequently, the manufacturers’ expectations of substantial profits rely on imposing high individual costs.

Despite presenting a curative potential, gene therapies must demonstrate health benefits. The design of the clinical studies is the main factor that generates uncertainty, because they are often performed with small sample sizes [7]. In addition, they are generally single-arm clinical trials and the follow-up is short [8]. This leads to a lack of demonstration of the efficacy and effectiveness of gene therapy, raising concerns about the sustainability of the long-term benefits beyond those demonstrated in clinical trials [9].

In order to ensure timely access to patients, it is critical that countries develop pricing and reimbursement strategies/models that continue to incentivize research and development without compromising the sustainability of healthcare systems. Therefore, payers and manufacturers need to acknowledge each other’s constraints and embrace innovative approaches to ensure timely delivery of therapies to patients [10]. This study aimed to review the available strategies that have been implemented or proposed for the coverage or reimbursement of gene therapies, as well as providing their main characteristics and barriers of use.

## Methods

### Aim

To review the evidence about strategies implemented or proposed for coverage or reimbursement for currently approved gene therapies across the world.

### Study design

A scoping review was conducted with the aim of comprehensively identifying the strategies implemented or proposed for the coverage or reimbursement of gene therapies across the world. The review was conducted following the methodology of the Joanna Briggs Institute (JBI) [11]. We adhered to the guidance of the Preferred Reporting Items for Systematic Reviews and MetaAnalyses (PRISMA) guidelines [12]. Subsequently, the research questions were: What are the strategies or mechanisms for coverage and reimbursement of gene therapies? What are their characteristics and barriers to implement these strategies in the different healthcare systems?

### Databases and search strategy

PUBMED/MEDLINE and OVID/EMBASE were used to search from the earliest available dates until February 2023. The research was guided by three domains: the first related to gene therapy, the second to financing and reimbursement, and the third to health systems. These keywords were validated by obtaining their respective Medical Subject Headings (MeSH) for their application in PubMed. The databases were limited to Spanish and English. Grey literature was excluded for this searching. Search strategies for PubMed/MEDLINE and OVID/EMBASE databases are provided in the Appendix.

### Citation management

The citations were imported into the citation manager EndNote X9. Then, the duplicates were removed, preparing the non-duplicate citations for title/abstract screening.

### Eligibility criteria

We included the original and review research articles which were published in full text until February 2023. Only articles in English and Spanish language were included. Inclusion criteria were publications that described models implemented or proposed for the coverage or reimbursement of gene therapy drugs, including the barriers or limitations of these strategies. Articles that focused on a specific disease or treatment (e.g. spinal muscular atrophy, haemophilia, cancer, etc.) were excluded. Studies or reports on health technology assessments (HTA) or the cost-effectiveness of these technologies were also excluded. Finally, studies for which full access was not available were also excluded.

### Screening of citations

Two rounds of screening were conducted to select eligible studies. Initially, two researchers independently screened titles and abstracts. In the event of a disagreement, a discussion was held until a consensus was reached. Titles without an available abstract were included for full text review. The relevant studies were subjected to a second level of screening, where two researchers reviewed the articles in full text. Only the studies that met the eligibility criteria were included. Any disagreements were resolved through discussion between the researchers.

### Data extraction and presentation

The data of the articles considered relevant for this scoping review were the following: authors, name of the study, name of the journal, year of publication, jurisdiction of the article, type of study proposed/objective of the work, main results/conclusions of the study and proposed mechanism.

## Results

**Fig. 1:**
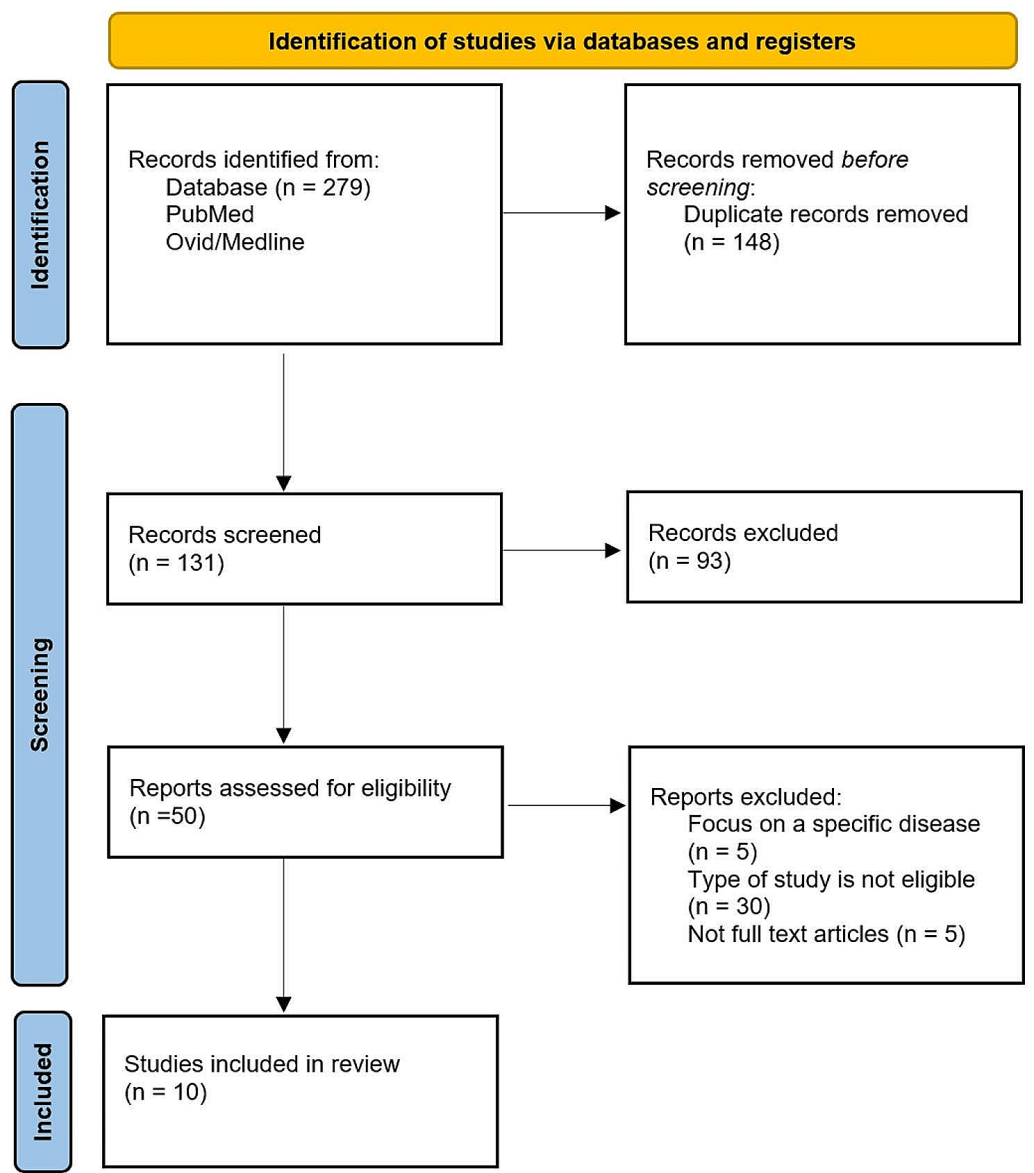
PRISMA flow diagram for the scoping review process

The literature search identified a total of 279 articles. After duplicate removal and screening titles and abstracts, 50 articles were eligible for full-text review. Of these, 40 were not considered since they did not meet the inclusion criteria (Fig. 1). Ten publications met the inclusion criteria and were selected for this scoping review.

### General characteristics of the literature

The articles were published between 2016 [13] and 2023 [14]. Most studies were published since 2019 [15–21]. One study was published in 2016 [13], and another in 2018 [22] (Table 1).

**Table 1:** Characteristics of the studies selected in the review

*N*.º	Authors	Jurisdiction	Aim/purpose	Results /Conclusion	Proposed mechanism
1	Carr & Bradshaw, 2016.	Not specified	To analyse the coverage challenges that gene therapies present to healthcare and insurance systems.	It is proposed to seek financing alternatives for gene therapy drugs since the current reimbursement models are not adapted to this type of technology. The authors recommend annuity payments based on clinical results, which will allow rewarding innovation, distributing the cost, and limiting payers’ financial risk and uncertainty regarding these new products, considering that their clinical benefit may appear much later to the administration.	Payment by annuities.
2	Hampson et al., 2018.	USA	To describe the challenges and possible solutions for implementing gene therapy in the USA Health System.	Affordability is one of the main challenges identified for the implementation of gene therapy. Among the solutions identified are risk-sharing agreements, which make it possible to guarantee the return of money if the expected results are not achieved for the patient. One challenge in implementing such a model is collecting evidence of the outcomes. The second problem is agreeing on contractual issues, the definition of therapeutic “success” or “failure”, and, finally, what will or will not be paid. Payback is another alternative to the affordability of these technologies, as a series of smaller payments over a period time is allowed. Certain characteristics were identified that will make certain gene therapies better candidates for this process, such as being a single treatment or having a curative clinical impact in the short term, the durability of clinical benefit that is well established or can be controlled through an outcome-based mechanism, a sufficiently large population size, among others.	Amortisation Outcome-Based Payment Models.
3	Barlow et al., 2019.	USA.	Explore feedback from healthcare payers regarding awareness of new gene therapies, the sustainability of current funding mechanisms, and the need for and preference for new funding models.	A high percentage of payers supported the new payment models, specifically performance-based agreements and risk-sharing agreements. Among the main challenges are the uncertainty related to the use of resources, the costs of new technologies and the duration of the clinical benefit. Payers cited regulation, plan rotation, and the ability to track long-term results as the main barriers to implementing the new financing models.	Performance-based agreements.
4	Ballreich et al.[16].	USA	Medicaid financing alternatives for gene therapies.	Use innovative financing models for these medicines, such as a Pooled Subscription-Based Model, which should be based on periodic evaluations regarding treatment efficacy and negotiation, jointly with all states, of a fixed fee for access to a or multiple therapies.	Model Based on pooled-subscription or “Netflix” type models.
5	Jørgensen et al., 2020 [18].	France Germany Italy Spain UK	To review the reimbursement and financing mechanisms used by 5 European countries for two advanced therapy drugs, identifying possible challenges in their implementation.	These two drugs were financed by the five countries analysed. However, the reimbursement mechanisms differed between each country: In one country (UK), the reimbursement is made through a national fund. An agreement for updated evidence is made to make a decision and reassess prices. In other countries (Spain and Italy), paying in instalments associated with the clinical results in the patients in which the innovative drug is administered was proposed. These deadlines were defined between patients, insurers and the industry. Additionally, challenges regarding the implementation of payment for results are pointed out. In effect, the creation of computer systems for monitoring patients is needed. Another challenge concerns healthcare facilities and human capital resources, which are needed to optimize patient results.	Outcome-Based Payment Models.
6	van Overbeeke et al., 2021	Canada USA Europe	To identify the main challenges in gene therapy implementation and propose solutions to this.	Reimbursement through outcome-based payment was proposed. The challenges for implementing these alternatives must be associated with modifying the laws and regulations that prevent their application in some cases. Additionally, infrastructure for data and real-world evidence collection is needed.	Outcome-Based Payment Models.
7	Jørgensen & Kefalas, 2021.	France Germany Italy Spain UK USA	To review innovative outcome-based reimbursement utilization schemes and payment mechanisms in gene therapy in USA and Europe.	Diverse coverage mechanisms have been found in each Europe country. France preliminarily covered Zynteglo® (gene therapy drug) with a data collection update condition for reassessing the technology in the next years. Gene therapy has been reimbursed under data collection conditions in Germany and France. In addition, Zongelsma® has been covered by outcome-based discounts linked to individualised patient data. Italy has used a reimbursement mechanism associated with results in three gene therapy drugs. These mechanisms are associated with instalment payments based on defined results. The Italian Medicines Agency is in charge of collecting the associated clinical data. Two gene drugs are covered through a reimbursement payment mechanism associated with results in Spain. This country has developed data collection and management systems to reduce new therapies’ uncertainty. The UK has covered gene therapy through an agreement with evidence updates for reassess in the next years. An exclusive fund finances these drugs. No established payment model among all payers exists in USA. There are experiences in outcome-based payment in some insurers for certain treatments.	Outcome-Based Payment Models Payment conditioned to Evidence.
8	Dabbous et al. [20]	Not specified	To analyse the feasibility of applying amortisation as a tool for the financing of gene therapy.	Given that gene therapies are an intangible asset, amortisation will allow the payer (or financing healthcare system) to cope with and mitigate the budget impact of gene therapy. This is through spreading its price over several years rather than absorbing it in the first year of administration. Thus, facilitating access to these products for new patients is available. However, several limitations need to be addressed. One of them is related to the uncertainty of potential long-term savings. Finally, the authors state that amortisation can be used with another innovative payment mechanism. A competent body (health technology assessment agency, for example) should define the use of these agreements.	Amortisation.
9	Koleva-Kolarova et al., 2022 [14].	Europe	To review the methods of financing and reimbursement of personalized medicine currently used in certain health systems. To know their characteristics and describe their process.	The authors analysed financing and reimbursement models applied in healthcare systems. Among the most used reimbursement models are risk-sharing arrangements based on therapeutic outcomes (coverage conditional on evidence generation, outcome-based discounts, annuity payment, and personalised performance-based reimbursement systems). Other countries have used purely financial risk-sharing arrangements, such as discounts, free therapy cycles, volume-based arrangements, pricing, and, most innovatively, “Netflix” type arrangements. Outcomes-based payment mechanisms could facilitate access to health technologies earlier. Barriers to the need for systems to collect data and measure clinical outcomes were pointed out. Other barriers were related to implementation problems due to the lack of accessible endpoints and administration costs.	Outcome-Based Payment Models “Netflix” type models.
10	Lee S & Lee JH, 2023	South Korea Europe	To capture the salient regulatory features of the cell and gene therapy market in the context of South Korea and the European Union	Different thresholds are available for innovative drugs in South Korea. Cost-effectiveness analysis can be exempted in cases of gene therapy and expenditure-capped risk sharing agreement becomes effective. In some cases, outcome-based agreement is proposed. Additionally, the lowest A7 countries adjusted prices is used for reference price. Barriers are related by challenge in generating clinical data for pharmacoeconomic studies as well for clinical necessities. Coverage with evidence development is applied in UK, France, and Germany. In other hand, outcome-based reimbursement (payment by results) is used in Italy, Germany, and Spain. In some cases, budget cap or ceiling cap are applied in Italy and Spain.	Expenditure-capped risk sharing agreement Outcome-based agreement

According to the jurisdiction of the selected papers, five indicated strategies proposed or developed for the United States of America (USA) [15, 16, 18, 19, 22]; five in Europe (France, Italy, Germany, Spain, United Kingdom) [14, 17–19, 21]; one in Canada [18]; one in South Korea [22], and two did not specify the country [13, 20].

### Aim and scopes

Eight studies were literature reviews [13, 14, 17–22], reporting experiences on various reimbursement schemes and their characteristics for implementation. Specifically, three studies analysed the main challenges of addressing the coverage of gene therapy drugs in health systems and identified possible solutions [13, 18, 22]. Dabbous et al. [20] studied the feasibility of applying amortisation as a tool to finance gene therapy from an accounting point of view, proposing this strategy as a new innovative payment mechanism [20]. The other four articles described reimbursement and coverage strategies applied to innovative drugs. These studies explained the virtues and challenges of their mechanism in each country analysed [14, 17, 19, 21].

Additionally, one study conducted qualitative interviews with different payers of the USA to have a better understanding of their knowledge of new gene therapies, the sustainability of current financing mechanisms, and the need and preference for a new model of financing [15]. Finally, Ballreich et al. [16] presented various alternatives applied to Medicaid to finance gene therapies [16].

### Proposals or mechanisms implemented for financing and coverage of gene therapy

#### Subscription-based payment models

Two studies proposed using subscription-based payment models known as “Netflix-like models” [16, 21]. In their study, Koleva-Kolarova et al. (2022) [21] defined this strategy as “a model based on the payment of a lump sum by the health system to the manufacturers in exchange for unlimited access for patients during a defined period”. However, the authors noted that it was unclear how the payment of these therapies would be implemented in practice, as the subscription fee, the uncertainty of expected results, and the duration of the subscription must be defined.

Ballreich et al. [16] suggested a potential strategy for the implementation of Medicaid in the USA. Specifically, the authors proposed that states could form a unified front to negotiate long-term contracts with manufacturers, enabling patients who satisfy selection criteria to access medications at a fixed price. This approach has the potential to be extended to a broader range of therapies, thereby enhancing scalability [16].

### Outcome-based payment models

Outcome-based payment models were identified in seven articles [14, 15, 17–19, 21, 22]. Koleva-Kolarova et al. (2022) characterised these mechanisms as coverage conditioned to the generation of evidence, discounts based on results, payment in annuities, and personalised reimbursement systems based on performance, among others [21]. The authors indicate that these contracts can be short-term (one year) or long-term (multi-year), with advance payments or in instalments based on agreed milestones. This agreement reduces the financial risk for payers in case of treatment failure or poor performance by sharing the costs with producers [21]. Currently, these types of agreements have been used in European countries, more specifically in Italy and Spain, through payment in instalments associated with outcomes for the coverage of advanced therapy drugs and gene therapies, such as Kymriah®, Yescarta®, Luxturna® and Zolgensma® [14, 17, 19]. Barlow et al. (2019) reported that 47% of payers support the implementation of new payment models, especially performance-based arrangements, and risk pooling [15].

Several barriers to those agreements have been identified in the literature. These include deficiencies in computer systems for data collection to measure clinical outcomes [14, 17, 18, 21, 22], the need to define “success” or “failure” of therapies to determine what will or will not be reimbursed [22], changes in laws or regulations that impede the adoption of these strategies [14, 18], administrative costs and the lack of readily accessible clinical endpoints [21].

### Amortisation

Amortisation was suggested as a financing strategy for gene therapies in two studies [20, 22]. This strategy can be defined as a key accounting principle that spreads the cost of an intangible asset over the periods in which a commercial organisation or entity receives the benefits of the asset [23].

Dabbous et al. [20] assessed the feasibility of amortisation as an accounting tool for gene therapy. The authors stated that this strategy might be a promising method to finance new health technologies. However, budget sustainability, health technologies eligibility, and financial regulations were identified as limitations and barriers. Hampson et al. (2018) identified some attributes that might make certain gene therapies better candidates for amortisation [22]. These attributes included: being a single treatment or having a short-term curative clinical impact, the durability of clinical benefit that is well established or can be controlled through an outcome-based mechanism, and sufficiently large population size [22].

## Discussion

This scoping review aimed to identify the main strategies proposed or implemented for the financing of gene therapy in various countries in the published literature. The results show that the main strategies applied have been in high-income countries. Most of the studies identified in this review focused on gene therapy to treat rare diseases, as gene therapies approved by regulatory agencies to date are for treating this type of conditions [24]. However, the identified strategies might apply to other types of gene therapy, such as those for cancer treatments.

Subscription-based payment models, also known as “Netflix-like models” were recommended by two studies. These models have been implemented in Australia and USA to fund the treatment against hepatitis C (HCV) [25–27]. This subscription-based payment models resulted in significant cost savings and increased access to HCV treatment [26]. Since 2022, the NHS England has used this strategy to fund antibiotics [28]. The implementation of this model in the NHS England has the potential to reduce costs and improve patient outcomes related to antibiotic resistance [28, 29].

European countries have applied outcome-based payment models for coverage of advanced therapies, including gene therapy [17, 19]. Also, in the USA, some payers have supported the use of such measures [29]. These types of agreements seek to reduce the first-order uncertainty surrounding the effectiveness (and potentially cost-effectiveness) of a product at individual level [30–32] by transferring responsibility to manufacturers for its results in the real world after its regulatory approval [33]. While these models may be attractive to facilitate early access to these expensive therapies facing uncertain results at patient level, they also face implementation barriers. They include the need for institutional capacity to monitor follow-up and assign outcomes, coordination among insurers in the context of multi-payer systems, and the need for covering implementation and transaction costs [34–37].

The literature shows some evidence of dealing with the challenge of data collection for monitoring. Spain’s National Health Service implemented Valtermed, a clinical data collection and management system, to reduce uncertainty related to outcome-based models for access to new medicines [38, 39]; whereas, in Italy, establishing and managing such data has shown to be underestimated in costs, and the actual amount reimbursed by the companies is negligible [40, 41]. Defining the governance of managing these models is crucial, whether by a state health authority or by autonomous entities, such as private or mixed organizations, in either a single or multi-payer system, remains as one of the major challenges.

Another type of access scheme analysed was the use of amortization as a financial strategy for the distribution of costs of intangible assets, such as gene therapies [20]. This strategy would allow systems to distribute the costs of the treatment over the period in which the patient receives the benefits, reducing the economic pressure on the health system and facilitating access to these drugs for patients [20]. To implement this strategy, gene therapies must be declared as intangible assets, as they provide a health benefit rather than a physical product [20, 22]. Financial regulations of countries would need to be modified to apply amortization in gene therapy, and only treatments of a single administration or short term with long-term benefits would be eligible [20]. However, further research is needed prior to implementing this strategy for gene therapies. This would require a deeper understanding of the benefits and drawbacks of amortization as a financial tool for these types of drugs.

The fact that the evidence comes from high income countries may be explained because strategies applied in low and middle-income countries have yet to be published in indexed journals, or because they have been recently implemented. This is, for example, the case of Zolgensma® in Argentina, where the federal government led to the development of an outcome-based risk share agreement with Novartis, which was recently informed in February 2023 [42, 43]. In this case, the payment was made in four installments over a 3-year period. Then, each instalment would be conditional on the fulfilment of certain outcomes agreed between both Novartis and the government. Another recent case study is Brazil where Zolgensma® was also covered through instalments payments linked with outcomes based on clinically agreed milestones [44].

The main limitation of our scoping review is that we may not have comprehensively captured all available published literature, given our search was restricted to two databases and literature published in English and Spanish, and we did not capture data published in the grey literature. However, we argue that most scientific reports across the globe have been published in one of these two languages. The timeframe of our search, that may also be considered a limitation, is reasonable given it starts when the first gene therapy was launched into the market.

Gene therapy is in a continuous process of development [45–48]. In fact, a report published by PhRMA listed almost 300 gene and cell therapies under investigation to treat several diseases [49]. More than one hundred of these treatments are focused on different types of cancer [49]. The identification of new targets is promising to treat rare diseases and improve existing cancer treatments [50, 51]. The development of new, more precise and specific gene editing techniques is highlighted, such as CRISPR, which could allow efficient modifications in the genome to treat cancer and other diseases [52, 53]. Undoubtedly, the growing innovation in gene therapy will make it possible to meet numerous needs of a wide spectrum of diseases. Therefore, it is important to assess the different capacities that health systems have to facilitate sustainable access to such therapies.

## Conclusion

Gene therapies are treatments that make it possible to cure or treat rare diseases and certain cancers, which until recently, were intractable. However, their high cost makes access to patients difficult. Our scoping review shows the main models proposed to finance and cover these disruptive treatments by health systems. This review revealed that there are different alternatives to cover these therapies. Each proposed strategy has its characteristics and barriers that are overcome for its implementation. Finally, it is necessary to continue publishing the experiences of the use of these models to continue obtaining evidence of their use and obtaining data for their evaluation.

### Electronic supplementary material

Below is the link to the electronic supplementary material.

Supplementary Material 1

## Data Availability

All data generated or analysed during this study are included in this published article (and its additional files).

## Abbreviations

JBI: Joanna Briggs Institute
PRISMA: Preferred Reporting Items for Systematic Reviews and MetaAnalyses
PCC: Population, Concept, and Context
USA: United State of America
PhRMA: Pharmaceutical Research and Manufacturers of America

## References

[1] Han Q, Fu H, Chu X, Wen R, Zhang M, You T (2022). Research advances in treatment methods and Drug Development for Rare diseases. Front Pharmacol.

[2] National Cancer Institute. Gene Therapy. https://www.cancer.gov/publications/dictionaries/cancer-terms/def/gene-therapy. Accesed 31 May 2023.

[3] Shahryari A, Saghaeian Jazi M, Mohammadi S, Razavi Nikoo H, Nazari Z, Hosseini ES (2019). Development and clinical translation of approved gene Therapy products for Genetic disorders. Front Genet.

[4] Kerpel-Fronius S, Baroutsou V, Becker S, Carlesi R, Collia L, Franke-Bray B (2020). Development and Use of Gene Therapy Orphan drugs-ethical needs for a broader Cooperation between the Pharmaceutical Industry and Society. Front Med (Lausanne).

[5] Jørgensen J, Kefalas P (2017). Annuity payments can increase Patient Access to innovative cell and Gene Therapies under England’s Net Budget Impact Test. J Mark Access Health Policy.

[6] Muigai AWT (2022). Expanding Global Access to genetic therapies. Nat Biotechnol.

[7] Hanna E, Rémuzat C, Auquier P, Toumi M. Advanced Therapy Medicinal products: current and future perspectives. J Mark Access Health Policy. 2016;4. 10.3402/jmahp.v4.31036 PMC4846788 27123193

[8] Abou-El-Enein M, Hey SP. Cell and Gene Therapy Trials: Are We Facing an ‘Evidence Crisis’? EClinicalMedicine. 2019;7:13 – 4. 10.1016/j.eclinm.2019.01.015 PMC6537560 31193634

[9] Sharpe M, Barry J, Kefalas P (2021). Clinical adoption of Advanced therapies: challenges and opportunities. J Pharm Sci.

[10] Coyle D, Durand-Zaleski I, Farrington J, Garrison L, Graf von der Schulenburg J-M, Greiner W (2020). Hta Methodology and Value frameworks for evaluation and policy making for cell and gene therapies. Eur J Health Econ.

[11] Peters M, Godfrey C, McInerney P, Soares C, Khalil H, Parker D. Methodology for Jbi Scoping Reviews. 2015. pp. 1–24.

[12] Tricco AC, Lillie E, Zarin W, O’Brien KK, Colquhoun H, Levac D (2018). Prisma Extension for scoping reviews (Prisma-Scr): Checklist and Explanation. Ann Intern Med.

[13] Carr DR, Bradshaw SE (2016). Gene therapies: the challenge of Super-high-cost treatments and how to pay for them. Regen Med.

[14] Lee S, Lee JH. Cell and Gene Therapy Regulatory, Pricing, and Reimbursement Framework: With a Focus on South Korea and the Eu. 2023;11. 10.3389/fpubh.2023.1109873 PMC9998493 36908458

[15] Barlow JF, Yang M, Teagarden JR (2019). Are payers ready, willing, and able to provide Access to new durable gene therapies?. Value Health.

[16] Ballreich J, Ezebilo I, Sharfstein J (2020). Affording Genetic therapies in the Medicaid Program. JAMA Pediatr.

[17] Jørgensen J, Hanna E, Kefalas P (2020). Outcomes-based reimbursement for Gene therapies in Practice: the experience of recently launched Car-T cell therapies in major European countries. J Mark Access Health Policy.

[18] van Overbeeke E, Michelsen S, Toumi M, Stevens H, Trusheim M, Huys I (2021). Market Access of Gene therapies across Europe, USA, and Canada: challenges, trends, and solutions. Drug Discov Today.

[19] Jørgensen J, Kefalas P (2021). The use of innovative payment mechanisms for Gene Therapies in Europe and the USA. Regen Med.

[20] Dabbous M, Toumi M, Simoens S, Wasem J, Saal G, Wang Y (2022). Amortization of gene replacement therapies: a Health Policy Analysis exploring a mechanism for Mitigating Budget Impact of high-cost treatments. Health Policy.

[21] Koleva-Kolarova R, Buchanan J, Vellekoop H, Huygens S, Versteegh M, Mölken MR (2022). Financing and reimbursement models for Personalised Medicine: a systematic review to identify current models and future options. Appl Health Econ Health Policy.

[22] Hampson G, Towse A, Pearson SD, Dreitlein WB, Henshall C (2018). Gene Therapy: evidence, Value and Affordability in the us Health Care System. J Comp Eff Res.

[23] Mueller J, Amortization of Certain Intangible Assets Journal of Accountancy. 2004. https://www.journalofaccountancy.com/issues/2004/dec/amortizationofcertainintangibleassets.html. Accesed 04 April 2023.

[24] Papanikolaou E, Bosio A (2021). The Promise and the Hope of Gene Therapy. Front Genome Ed.

[25] Moon S, Erickson E. Universal Medicine Access through Lump-Sum Remuneration — Australia’s Approach to Hepatitis C. 2019;380(7):607–10. 10.1056/NEJMp1813728 30763190

[26] Matthews DW, Coleman S, Razavi H, Izaret J-M (2022). The payer license agreement, or Netflix Model, for Hepatitis C Virus therapies enables Universal Treatment Access. Lowers Costs Incentivizes Innov Competition.

[27] Gene Therapy’s Next Installment (2019). Nat Biotechnol.

[28] Mahase E (2020). Uk launches subscription style model for antibiotics to encourage New Development. J BMJ.

[29] Barlow E, Morton A, Megiddo I, Colson A (2022). Optimal subscription models to pay for antibiotics. Soc Sci Med.

[30] Espinoza MA, Manca A, Claxton K, Sculpher MJ (2014). The value of heterogeneity for cost-effectiveness subgroup analysis: conceptual Framework and Application. Med Decis Mak.

[31] Espinoza M, Manca A, Sculpher M, Claxton K (2012). Co1 individual decisions and Social Value: a conceptual Framework to explore alternative decision making approaches and the value of heterogeneity in the era of Individualized Care. Value Health.

[32] Espinoza MA, Sculpher MJ, Manca A, Basu A, Culyer AJ (2014). Analysing heterogeneity to support decision making. Encyclopedia of Health Economics.

[33] Dabbous M, Chachoua L, Caban A, Toumi M (2020). Managed Entry agreements: policy analysis from the European perspective. Value Health.

[34] Faulkner A, Mahalatchimy A (2018). The politics of valuation and payment for Regenerative Medicine products in the Uk. New Genet Soc.

[35] Michelsen S, Nachi S, Van Dyck W, Simoens S, Huys I (2020). Barriers and opportunities for implementation of outcome-based spread payments for High-Cost, one-shot curative therapies. Front Pharmacol.

[36] Simoens S, De Groote K, Boersma C (2022). Critical reflections on reimbursement and Access of Advanced therapies. Front Pharmacol.

[37] Clopés Estela A, Soler Rotllant F, Germà Lluch JR, Calle Rodríguez C (2022). Pay-for-performance schemes: 10 years’ experience in a Comprehensive Cancer Center. Med Clínica (English Edition).

[38] Ministerio de Sanidad, Preguntas Y. Secretaría general de Sanidad y Consumo. Dirección general de cartera básica de servicios del SNS y farmacia: Ministerio de Sanidad; 2019. Respuestas Frecuentes Sobre El Sistema De Información Para Determinar El Valor Terapéutico En La Práctica Clínica Real De Los Medicamentos De Alto Impacto Sanitario Y Económico En El Sistema Nacional De Salud (Valtermed).

[39] Diario Farma, Valtermed. La Conexión Y El Registro De Resultados Clínicos Ya Es Posible Diario Farma; 2019. https://diariofarma.com/2019/07/22/valtermed-la-conexion-y-el-registro-de-resultados-clinicos-ya-es-posible. Accesed 05 April 2023.

[40] Garattini L, Curto A, van de Vooren K (2015). Italian risk-sharing agreements on drugs: are they worthwhile?. Eur J Health Econ.

[41] Navarria A, Drago V, Gozzo L, Longo L, Mansueto S, Pignataro G (2015). Do the current performance-based schemes in Italy really work? Success Fee: a Novel measure for cost-Containment of Drug Expenditure. Value Health.

[42] Boletín Oficial de la República Argentina. Legislación Y Avisos Oficiales. Ministerio De Salud Subsecretaría De Medicamentos E Información Estratégica Argentina Presidencia. 2023. https://www.boletinoficial.gob.ar/detalleAviso/primera/279782/20230116. Accesed 02 May 2023.

[43] Ministerio de Salud. El Estado Garantiza El Acceso Al Tratamiento De La Atrofia Muscular Espinal (Ame) Mediante Una Estrategia De Riesgo Compartido Superintendencia de Servicios de Salud. 2023. https://www.argentina.gob.ar/noticias/el-estado-garantiza-el-acceso-al-tratamiento-de-la-atrofia-muscular-espinal-ame-mediante. Accesed 02 May 2023.

[44] Ministério da Saúde. Ms E Novartis Firmam Compromisso Para Elaboração Do Acordo De Compartilhamento De Risco Para Ame Comissão Nacional de Incorporação de Tecnologias no SUS (CONITEC). 2022. https://www.gov.br/conitec/pt-br/assuntos/noticias/2022/dezembro/ms-e-novartis-firmam-compromisso-para-elaboracao-do-acordo-de-compartilhamento-de-risco-para-ame. Accesed 05 April 2023.

[45] Zhang Q, Kuang G, Li W, Wang J, Ren H, Zhao Y. Stimuli-Responsive Gene Delivery Nanocarriers for Cancer Therapy. Nano-Micro Lett. 2023;15(1). 10.1007/s40820-023-01018-4 PMC9908819 36752939

[46] Gao TT, Oh TJ, Mehta K, Huang YA, Camp T, Fan H (2023). The clinical potential of Optogenetic Interrogation of Pathogenesis. Clin Transl Med.

[47] Kang L, Jin S, Wang J, Lv Z, Xin C, Tan C (2023). Aav vectors Applied to the treatment of Cns disorders: clinical Status and challenges. J Controlled Release.

[48] Mannucci PM (2023). Hemophilia Treatment Innovation: 50 years of Progress and more to come. J Thromb Haemost.

[49] PhRMA. Medicines in Development for Cell and Gene Therapy 2020 Pharmaceutical Research and Manufacturers of America. 2020. https://phrma.org/medicines-in-development/medicines-in-development-for-cell-and-gene-therapy-2020. Accesed 03 May 2023.

[50] Drakopoulou E, Anagnou NP, Pappa KI (2022). Gene Therapy for Malignant and Benign Gynaecological disorders: a systematic review of an emerging Success Story. Cancers.

[51] Muramatsu K, Muramatsu S-I (2023). Adeno-Associated Virus Vector-based Gene therapies for Pediatric diseases. Pediatr Neonatology.

[52] Onishi I, Yamamoto K, Kinowaki Y, Kitagawa M, Kurata M (2021). To Discover the efficient and novel drug targets in human cancers using Crispr/Cas screening and databases. Int J Mol Sci.

[53] Vaghari-Tabari M, Hassanpour P, Sadeghsoltani F, Malakoti F, Alemi F, Qujeq D et al. Crispr/Cas9 gene editing: a New Approach for Overcoming Drug Resistance in Cancer. Cell Mol Biol Lett. 2022;27(1). 10.1186/s11658-022-00348-2 PMC9204876 35715750

