# Supplementary material for: Challenges for gene therapy in the financial sustainability of health systems: a scoping review
Source: Orphanet J Rare Dis. 2024 Jun 24;19:243. doi: 10.1186/s13023-024-03249-z (PMC11197217; doi:10.1186/s13023-024-03249-z)
Supplement: Supplementary file 1 — Supplementary Material 1 [file 13023_2024_3249_MOESM1_ESM.docx]

***Appendix***

Search strategies

PUBMED/MEDLINE

|  | |  |
| --- | --- | --- |
| **1** | "Genetic Therapy"[MAJR] | n = 33.109 |
| **2** | Therap*[Title] (genetic [Title] OR DNA[Title] OR Gene [Title] OR RNAi[Title]) | n = 28.248 |
| **3** | Targeted [Title/Abstract] (Gene Repair*[Title/Abstract] OR Mutation Repair*[Title/Abstract] OR Gene Correction*[Title/Abstract]) OR "Personalised Medicine"[Title/Abstract] | n = 2.035 |
| **4** | #1 OR #2 OR #3 | n = 47.868 |
| **5** | "Costs and Cost Analysis"[MAJR] | n = 78.945 |
| **6** | "Cost-Benefit Analysis"[MAJR] | n = 10.211 |
| **7** | "Genetic Therapy/economics"[MAJR] | n = 149 |
| **8** | Cost [All fields] OR costs [All fields] OR economic [Title] OR "payment"[All Fields] OR "insurance, health, reimbursement/economics*"[MeSH Terms] OR "financing, government"[MeSH Major Topic] OR "pay-for-performance"[All Fields] OR "insurance, health, reimbursement"[MeSH Terms] OR "outcome-based agreement"[All Fields] OR "spread payment"[All Fields] OR "affordability"[All Fields] OR "Coverage"[All Fields] OR "Reimbursement Mechanisms"[MeSH Terms] OR "Policy Making"[MeSH Major Topic] OR "Managed Entry Agreements"[All Fields] OR "Performance-based agreements"[All Fields] OR "innovative payment mechanisms"[All Fields] OR "Outcomes-Based Managed Entry Agreements"[All Fields] OR "performance-based"[All Fields] OR "performance-based"[All Fields] OR "performance-linked"[All Fields] OR "performance-linked"[All Fields] OR "pay-for-performance"[All Fields] OR "pay-for-performance"[All Fields] OR "paying-for-performance"[All Fields] OR "paying-for-performance"[All Fields] OR "outcome-based"[All Fields] OR "outcome-based"[All Fields] OR "outcomes-based"[All Fields] OR "outcomes-based"[All Fields] OR "coverage with evidence"[All Fields] OR "access with evidence"[All Fields] OR "payment model"[All Fields] OR "payment models"[All Fields] OR "annuity"[All Fields] OR "annuities"[All Fields] OR "leasing"[All Fields] OR "lease"[All Fields] OR "rent"[All Fields] OR "renting"[All Fields] OR "amortization"[All Fields] OR "pooled budget"[All Fields] OR "pooled budgets"[All Fields] OR "combined budget"[All Fields] OR "reimbursement"[All Fields] OR "sustainable financing"[All Fields] OR "debt financing"[All Fields] OR "patient access"[All Fields] OR "managed entry"[All Fields] OR "managed access"[All Fields] OR "risk-sharing"[All Fields] OR "risk-sharing"[All Fields] OR "value-based"[All Fields] OR "value-based"[All Fields] | n = 1.355.340 |
| **9** | #5 OR #6 OR #7 OR #8 | n = 1.355.340 |
| **10** | "Genetic Therapy"[MAJR] AND "for all" [Title/Abstract] | n = 203 |
| **11** | "Delivery of Health Care/economics"[MAJR] | n = 29.153 |
| **12** | "Health Services Accessibility"[Mesh] | n = 123.372 |
| **13** | "health care systems" OR "healthcare systems" OR "health care system" OR "healthcare system" OR "insurance, health"[MeSH Terms] OR "Insurance Coverage"[MeSH Terms] OR "financing, government"[MeSH Terms] | n = 363.154 |
| **14** | #11 OR #12 OR #13 | n = 473.334 |
| **15** | #9 AND #14 | n = 217.708 |
| **16** | #4 AND #15 | n = 128 |
| **17** | #10 OR #16 | n = 329 |
| **18** | 2016/01/01:2022/05/31[Date - Publication] AND 2016/01/01:2022/05/31[Date - Publication] | n = 8.561.080 |
| **19** | #17 AND #18 | n = 131 |

(("Genetic Therapy"[MeSH Major Topic] AND "for all"[Title/Abstract]) OR (("Genetic Therapy"[MeSH Major Topic] OR ("therap*"[Title] AND ("genetic"[Title] OR "DNA"[Title] OR "Gene"[Title] OR "RNAi"[Title])) OR ("Targeted"[Title/Abstract] AND ("gene repair*"[Title/Abstract] OR "mutation repair*"[Title/Abstract] OR "gene correction*"[Title/Abstract] OR "Personalised Medicine"[Title/Abstract]))) AND (("genetic therapy/economics"[MeSH Major Topic] OR (("economics"[MeSH Subheading] OR "economics"[Title/Abstract] OR "payment"[Title/Abstract] OR "insurance, health, reimbursement/economics*"[MeSH Terms] OR "financing, government"[MeSH Major Topic] OR "pay-for-performance"[Title/Abstract] OR "insurance, health, reimbursement"[MeSH Terms] OR "outcome-based agreement"[Title/Abstract] OR "spread payment"[Title/Abstract] OR "affordability"[Title/Abstract] OR "Coverage"[Title/Abstract] OR "Reimbursement Mechanisms"[MeSH Terms] OR "Policy Making"[MeSH Major Topic] OR "Managed Entry Agreements"[Title/Abstract] OR "Performance-based agreements"[Title/Abstract] OR "innovative payment mechanisms"[Title/Abstract] OR “risk-sharing agreements” OR "Outcomes-Based Managed Entry Agreements"[Title/Abstract] OR ("performance-based"[Title/Abstract] OR "performance-based"[Title/Abstract] OR "performance-linked"[Title/Abstract] OR "performance-linked"[Title/Abstract] OR "pay-for-performance"[Title/Abstract] OR "pay-for-performance"[Title/Abstract] OR "paying-for-performance"[Title/Abstract] OR "paying-for-performance"[Title/Abstract] OR "outcome-based"[Title/Abstract] OR "outcome-based"[Title/Abstract] OR "outcomes-based"[Title/Abstract] OR "outcomes-based"[Title/Abstract] OR "coverage with evidence"[Title/Abstract] OR "access with evidence"[Title/Abstract] OR "payment model"[Title/Abstract] OR "payment models"[Title/Abstract] OR "annuity"[Title/Abstract] OR "annuities"[Title/Abstract] OR "leasing"[Title/Abstract] OR "lease"[Title/Abstract] OR "rent"[Title/Abstract] OR "renting"[Title/Abstract] OR "amortization"[Title/Abstract] OR "pooled budget"[Title/Abstract] OR "pooled budgets"[Title/Abstract] OR "combined budget"[Title/Abstract])) OR ("reimbursement"[Title/Abstract] OR "sustainable financing"[Title/Abstract] OR "debt financing"[Title/Abstract] OR "patient access"[Title/Abstract] OR "managed entry"[Title/Abstract] OR "managed access"[Title/Abstract] OR "risk-sharing"[Title/Abstract] OR "risk-sharing"[Title/Abstract] OR "value-based"[Title/Abstract] OR "value-based"[Title/Abstract]))) AND ("delivery of health care/economics"[MeSH Major Topic] OR "Health Services Accessibility"[MeSH Terms] OR "Health Care Costs"[MeSH Terms] OR ("health care systems"[Title/Abstract] OR "healthcare systems"[Title/Abstract] OR "health care system"[Title/Abstract] OR "healthcare system"[Title/Abstract] OR ”Medicaid”[Title/Abstract] OR "insurance, health"[MeSH Terms] OR "Insurance Coverage"[MeSH Terms] OR "financing, government"[MeSH Terms])))

OVID/EMBASE

| **#** | **Search** | **Results** |
| --- | --- | --- |
| 1 | Genetic Therapy/ | 52,610 |
| 2 | ((genetic or DNA or gene or RNAi) and therapy).mp. [mp=title, book title, abstract, original title, name of substance word, subject heading word, floating sub-heading word, keyword heading word, organism supplementary concept word, protocol supplementary concept word, rare disease supplementary concept word, unique identifier, synonyms, population supplementary concept word, anatomy supplementary concept word] | 529,403 |
| 3 | (Targeted or repair or mutation repair or gene correction or personalised medicine).mp. [mp=title, book title, abstract, original title, name of substance word, subject heading word, floating sub-heading word, keyword heading word, organism supplementary concept word, protocol supplementary concept word, rare disease supplementary concept word, unique identifier, synonyms, population supplementary concept word, anatomy supplementary concept word] | 856,935 |
| 4 | 1 or 2 or 3 | 1,309,058 |
| 5 | (Costs or costs analysis or costs of illness).mp. [mp=title, book title, abstract, original title, name of substance word, subject heading word, floating sub-heading word, keyword heading word, organism supplementary concept word, protocol supplementary concept word, rare disease supplementary concept word, unique identifier, synonyms, population supplementary concept word, anatomy supplementary concept word] | 327,353 |
| 6 | Cost-Benefit Analysis/ | 91,970 |
| 7 | (Genetic Therapy and economics).mp. [mp=title, book title, abstract, original title, name of substance word, subject heading word, floating sub-heading word, keyword heading word, organism supplementary concept word, protocol supplementary concept word, rare disease supplementary concept word, unique identifier, synonyms, population supplementary concept word, anatomy supplementary concept word] | 503 |
| 8 | (cost or costs or economic or payment or insurance health or reimbursement or financing or government or pay-for-performance or outcome-based agreement or spread payment or affordability or coverage or Reimbursement Mechanisms or Policy Making or Managed Entry Agreements or Performance-based agreements or innovative payment mechanisms or Outcomes-Based Managed Entry Agreements or performance-based or performance-linked or pay-for-performance or paying-for-performance or outcome-based or coverage with evidence or access with evidence or payment model or payment models or annuity or annuities or leasing or lease or rent or renting or amortization or pooled budget or pooled renting or pooled budgets or combined budget or reimbursement or sustainable financing or debt financing or patient access or managed entry or managed access or risk-sharing or value-based or value).mp. [mp=title, book title, abstract, original title, name of substance word, subject heading word, floating sub-heading word, keyword heading word, organism supplementary concept word, protocol supplementary concept word, rare disease supplementary concept word, unique identifier, synonyms, population supplementary concept word, anatomy supplementary concept word] | 2,686,552 |
| 9 | (Genetic therapy and for all).mp. [mp=title, book title, abstract, original title, name of substance word, subject heading word, floating sub-heading word, keyword heading word, organism supplementary concept word, protocol supplementary concept word, rare disease supplementary concept word, unique identifier, synonyms, population supplementary concept word, anatomy supplementary concept word] | 5,622 |
| 10 | 5 or 6 or 7 or 8 | 2,686,811 |
| 11 | "Delivery of Health Care"/ | 114,723 |
| 12 | Health Services Accessibility/ | 84,928 |
| 13 | (Health care systems or insurance health or Insurance Coverage or financing or government).mp. [mp=title, book title, abstract, original title, name of substance word, subject heading word, floating sub-heading word, keyword heading word, organism supplementary concept word, protocol supplementary concept word, rare disease supplementary concept word, unique identifier, synonyms, population supplementary concept word, anatomy supplementary concept word] | 279,980 |
| 14 | 11 or 12 or 13 | 444,749 |
| 15 | 10 and 14 | 310,434 |
| 16 | 4 and 15 | 7,214 |
| 17 | 9 or 16 | 12,800 |
| 18 | limit 17 to (abstracts and full text and yr="2016 - 2022") | 2,721 |
| 19 | limit 18 to ovid full text available | 148 |
